# Enhanced group II mGluR-mediated inhibition of pain-related synaptic plasticity in the amygdala

**DOI:** 10.1186/1744-8069-2-18

**Published:** 2006-05-08

**Authors:** Jeong S Han, Yu Fu, Gary C Bird, Volker Neugebauer

**Affiliations:** 1 Department of Neuroscience and Cell Biology, The University of Texas Medical Branch, Galveston, Texas 77555-1069, USA

## Abstract

**Background:**

The latero-capsular part of the central nucleus of the amygdala (CeLC) is the target of the spino-parabrachio-amygdaloid pain pathway. Our previous studies showed that CeLC neurons develop synaptic plasticity and increased neuronal excitability in the kaolin/carrageenan model of arthritic pain. These pain-related changes involve presynaptic group I metabotropic glutamate receptors (mGluRs) and postsynaptic NMDA and calcitonin gene-related peptide (CGRP1) receptors. Here we address the role of group II mGluRs.

**Results:**

Whole-cell current- and voltage-clamp recordings were made from CeLC neurons in brain slices from control rats and arthritic rats (>6 h postinjection of kaolin/carrageenan into the knee). Monosynaptic excitatory postsynaptic currents (EPSCs) were evoked by electrical stimulation of afferents from the pontine parabrachial (PB) area. A selective group II mGluR agonist (LY354740) decreased the amplitude of EPSCs more potently in CeLC neurons from arthritic rats (IC_50 _= 0.59 nM) than in control animals (IC_50 _= 15.0 nM). The inhibitory effect of LY354740 was reversed by a group II mGluR antagonist (EGLU) but not a GABA_A _receptor antagonist (bicuculline). LY354740 decreased frequency, but not amplitude, of miniature EPSCs in the presence of TTX. No significant changes of neuronal excitability measures (membrane slope conductance and action potential firing rate) were detected.

**Conclusion:**

Our data suggest that group II mGluRs act presynaptically to modulate synaptic plasticity in the amygdala in a model of arthritic pain.

## Background

The amygdala plays a key role in the emotional processing of sensory stimuli [1-3]. Pain has a strong emotional component and is significantly associated with affective disorders such as depression and anxiety [4]. Accumulating evidence suggests that the amygdala is a neural substrate of the reciprocal relationship between pain and affect [5]. It has become clear now that lesions and pharmacological deactivation of the amygdala produce inhibitory effects on pain behavior in animals [6-8]. Several neuro-imaging studies have repeatedly identified pain-related signal changes in the amygdala in animals and humans [8-12].

The amygdala contains several anatomically and physiologically distinct nuclei. The central nucleus of the amygdala (CeA) is of particular interest because of its morphological and functional characteristics. Neurons in the latero-capsular part of the CeA (CeLC) receive relatively unprocessed nociceptive information directly (not involving thalamus and/or cortex) through the spino-parabrachio-amygdaloid pain pathway arising from lamina I neurons in the spinal cord [5,13,14] Direct spino-amygdaloid connections from spinal neurons in deeper laminae may also provide nociceptive information to the CeA [15]. The CeLC receives highly processed polymodal information with affective valence from the lateral and basolateral nuclei of the amygdala, the center of the fear-anxiety circuitry [16]. The CeA is the major output nucleus of the amygdala and projects to a variety of "upstream" and "downstream" targets that are involved in emotional behavior and emotional experience, autonomic and somatomotor functions and endogenous pain control [1,5,13,16,17].

The CeLC is now defined as the "nociceptive amygdala" because of its high content of nociceptive neurons [5]. Previous *in vivo *studies showed that the majority of CeLC neurons, including multireceptive and normally non-responsive neurons, are sensitized to sensory inputs after the induction of arthritic pain in one knee [8,18-20]. The sensitization consists of increased background activity, stronger activation by constant electrical stimulation of afferent inputs, and enhanced responses to brief noxious and innocuous stimulation (compression) of the arthritic knee and of non-injured tissue. Parallel *in vitro *whole-cell patch-clamp recordings in brain slices indicated that synaptic transmission to the CeLC is facilitated in the arthritis pain model [8,21-23]. Excitability of CeLC neurons is also enhanced in brain slices from arthritic rats.

The mechanisms of pain-related plasticity in the amygdala are only beginning to emerge, but glutamate receptors appear to be of critical importance. Metabotropic glutamate receptors (mGluRs) form a family of G-protein coupled receptors and have been implicated in neuroplasticity associated with normal brain functions as well as in a variety of nervous system disorders [24,25]. It is clear now that mGluRs also play an important role in nociception and pain [26-30]. Eight mGluR subtypes have been cloned to date and are classified into groups I (mGluR1,5), II (mGluR2,3) and III (mGluR4,6,7,8). Group I mGluRs couple to the activation of phospholipase C, resulting in calcium release from intracellular stores and protein kinase C (PKC) activation. In contrast, groups II and III mGluRs are negatively coupled to adenylyl cyclase, thereby inhibiting cyclic AMP (cAMP) formation and cAMP-dependent protein kinase (PKA) activation.

Our previous studies showed that arthritis pain-related sensitization and synaptic plasticity in the CeLC depend on presynaptic group I mGluR upregulation [19,21] and on postsynaptic N-methyl-D-aspartate (NMDA) receptor phosphorylation through the cAMP-dependent protein kinase PKA [20,22]. PKA activation is accomplished through postsynaptic calcitonin gene-related (CGRP1) receptors [8]. Conversely, activation of presynaptic group III mGluRs inhibited pain-related synaptic plasticity in the CeLC [23].

Here we analyze the role of group II mGluRs in pain-related plasticity in the CeLC. The rationale is as follows. 1) Group II mGluRs couple to the inhibition of stimulated cAMP formation, and cAMP-dependent PKA plays an important role in pain-related plasticity in the CeLC [5,20,22]. 2) There is evidence to suggest that group II mGluRs on primary afferents, in the spinal cord and brainstem modulate nociceptive processing but the role group II mGluRs in higher brain centers in prolonged or chronic pain states remains to be determined [26,27,29,30]. 3) Finally, potential clinical indications for group II mGluR agonists include anxiety disorders [24,31], which critically involve the amygdala; the reciprocal relationship between pain and anxiety is well documented [5].

## Results

### A selective group II mGluR agonist (LY354740) inhibits pain-related synaptic plasticity more potently than normal synaptic transmission

Our previous studies showed that CeLC neurons undergo several neuroplastic changes in the kaolin/carrageenan mono-arthritis pain model [8,21-23]. These changes include enhanced input-output functions of synaptic transmission at the PB-CeLC synapse (part of the spino-parabrachio-amygdaloid pain pathway [see [5]]), enhanced excitability and altered intrinsic membrane properties such as resting membrane potential, input resistance, membrane slope conductance and action potential threshold. These observations indicate synaptic and neural plasticity because arthritis pain-related changes are preserved in the reduced slice preparation and maintained, at least in part, independently of peripheral and spinal pain mechanisms [see [5]].

**Figure 1:**
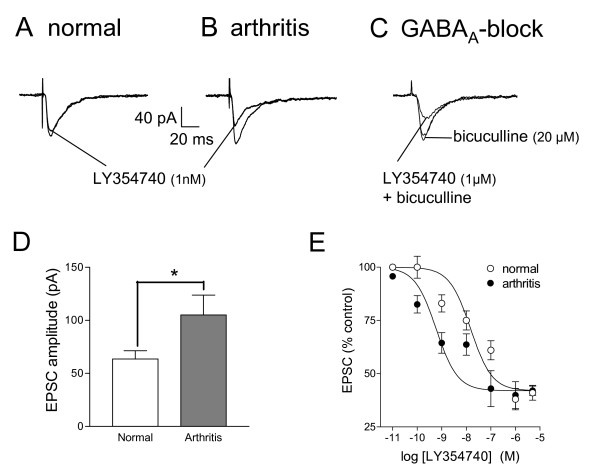
**Increased inhibitory potency of a group II mGluR agonist in the arthritis pain model**. **A**: A selective group II mGluR agonist (LY354740; 1 nM, 12 min) has little inhibitory effect in a CeLC neuron recorded in a slice from a normal rat. **B**: In a CeLC neuron from an arthritic rat, LY354740 (1 nM, 12 min) strongly decreases the EPSC amplitude. **A, B**: Each trace is the average of 10 monosynaptic EPSCs evoked at the PB-CeLC synapse and recorded in whole-cell voltage-clamp at -60 mV (see Methods). **C**: LY354740 (1 μM) inhibited synaptic transmission in a CeLC neuron from a normal rat in the presence of bicuculline (20 μM) to block GABA_A _receptors. Bicuculline alone had no apparent effect. **D**: The mean EPSC amplitude of CeLC neurons from arthritic rats (n = 13) was significantly higher compared to neurons from normal rats (n = 12, unpaired t-test), suggesting enhanced synaptic transmission (synaptic plasticity). **E**: Cumulative concentration-response relationships (see Methods) show that LY354740 inhibits synaptic transmission in CeLC neurons from arthritic rats more potently than in control neurons from normal rats (P < 0.01, F_1,45 _= 7.89, two way ANOVA). IC_50_s are 15.0 nM (normal) and 0.59 nM (arthritis). Peak amplitudes of EPSCs recorded at the PB-CeLC synapse during each concentration of LY354740 in neurons from normal rats (n = 9) and neurons from arthritic rats (n = 13) were averaged and expressed as percent of predrug (baseline) control (100%). LY354740 was applied by superfusion of the slice in ACSF for 12 min. Symbols and error bars represent mean ± SE. IC_50 _values were calculated as described in Methods. * P < 0.05.

In the present study, monosynaptic excitatory synaptic currents (EPSCs; see Methods) were recorded in neurons of the latero-capsular division of the central nucleus of the amygdala (CeLC) in brain slices from normal rats (n = 9 neurons) and from arthritic rats (6 h postinjection of kaolin/carrageenan into the knee; n = 13 neurons). Under normal conditions, a selective group II mGluR agonist (LY354740, 1 nM) [24,31] slightly inhibited the peak amplitude of monosynaptic EPSCs evoked at the PB-CeLC synapse (see individual example in Fig. 1A). In brain slices from arthritic rats, the same low concentration of LY354740 (1 nM) produced stronger inhibition of synaptic transmission (see individual example in Fig. 1B). The inhibitory effects of LY354740 did not involve GABAergic mechanisms since they were not blocked by bicuculline (20 μM; Fig. 1C). CeLC neurons in brain slices from arthritic rats showed significantly increased synaptic transmission (n = 12, unpaired t-test; Fig. 1D), which is consistent with our previous studies [8,21-23]. Fig. 1D shows the significantly increased peak EPSC amplitudes of neurons in slices from arthritic animals. Our previous studies determined that this measure reliably reflects altered input-output function at the PB-CeLC synapse [8,21-23]. Analysis of the cumulative concentration-response relationships (Fig. 1E; see Methods) showed a 25-fold increase in potency of LY354740 in the arthritis pain model (EC_50 _= 0.59 nM, n = 13; Fig. 1E, filled circles) compared to compared t normal conditions (EC_50 _= 15.0 nM, n = 9; Fig. 1E, open circles). The leftward shift of the LY354740 concentration-response function functio in the arthritis pain model was significant (P < 0.01, F_1,45 _= 7.89, two way ANOVA). Differences between concentrations were highly significant (P < 0.0001; F _6,45 _= 26.92). Consistent with the parallel shift of the concentration-response curves, there was no significant interaction (P > 0.05; F _6,45 _= 1.01).

### A selective group II mGluR antagonist (EGLU) reverses the effects of LY354740

**Figure 2:**
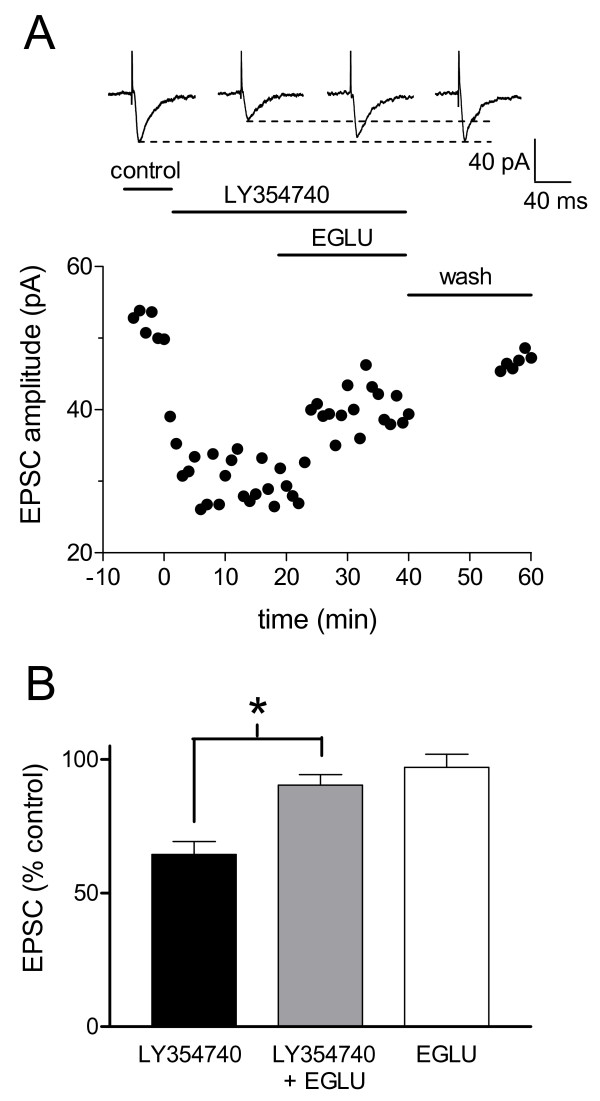
**A selective group II mGluR antagonist (EGLU) reverses the inhibitory effect of LY354740**. **A**: Whole-cell voltage-clamp recordings of an individual CeLC neuron in a brain slice from an arthritic rat show that LY354740 (10 nM) alone decreases the EPSC amplitude. This synaptic inhibition is largely reversed during co-administration of EGLU (10 μM). Following washout (>15 min), synaptic strength (peak amplitude) returns to baseline. **B**: EGLU (10 μM, 10–12 min) has no effect on EPSCs when applied alone (P > 0.05, Mann-Whitney U-test, n = 7) but reverses inhibition induced by LY354740. In the presence of EGLU (10 μM) the inhibitory effects of LY354740 (10 nM) are significantly attenuated (P < 0.05, paired t-test, n = 5). Inhibition of EPSC amplitude is expressed as the percentage of predrug controls (mean ± SE). * P < 0.05.

A selective group II mGluR antagonist (EGLU, 10 μM) [25,32] was used to verify receptor-mediated effects of LY354740 and to determine any intrinsic or endogenous receptor activation. Fig. 2A shows the time course of the effects of successive applications of LY354740 and EGLU coapplied with LY354740 in an individual CeLC neuron from an arthritic animal. In the sample of CeLC neurons from arthritic rats (n = 7; Fig. 2B), LY354740 alone (10 nM, 10 min) suppressed the EPSC amplitude to 64.4% ± 4.82 of the predrug value. The inhibitory effect of LY354740 was significantly attenuated (P < 0.05, paired t-test, n = 5) by the co-application of LY354740 (10 nM) and (EGLU; 10 μM, 10–12 min). EGLU alone (10 μM, 10–12 min) had no effect on synaptic transmission in CeLC neurons from arthritic rats (97.0% ± 4.85 of predrug control, n = 7; Fig. 2B).

### LY354740 acts pre- rather than postsynaptically in the CeLC

**Figure 3:**
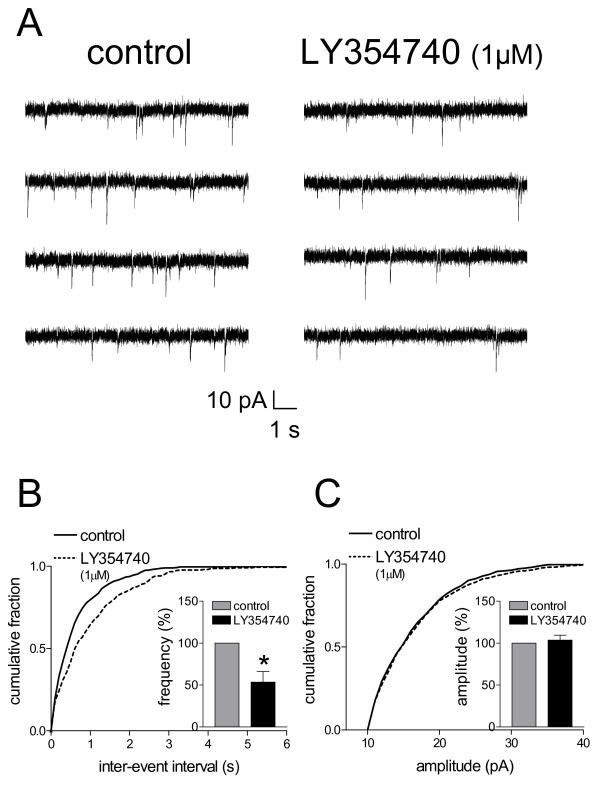
**Miniature EPSC (mEPSC) analysis indicates pre- rather than post-synaptic effects of LY354740**. **A**: Original current traces of mEPSC recorded in an individual CeLC neuron in the presence of TTX (1 μM) show that LY354740 (1 μM) reduces frequency but not amplitude of mEPSCs. The CeLC neuron was recorded in a slice from an arthritic rat. **B, C**: Normalized cumulative distribution analysis of mEPSC amplitude and frequency shows that LY354740 causes a significant shift toward higher inter-event intervals (decreased frequency) (**B**, P < 0.005, maximal difference in cumulative fraction = 0.183, Kolmogorov-Smirnov test) but had no effect on the amplitude distribution (**C**). LY354740 selectively decreased mean mEPSC frequency (events/s) (P < 0.05, paired t-test) but not mEPSC amplitude in the sample of neurons (n = 4; see bar histograms in **B, C**). Symbols and error bars represent mean ± SE. Neurons were recorded in voltage-clamp at -60 mV. * P < 0.05.

**Figure 4:**
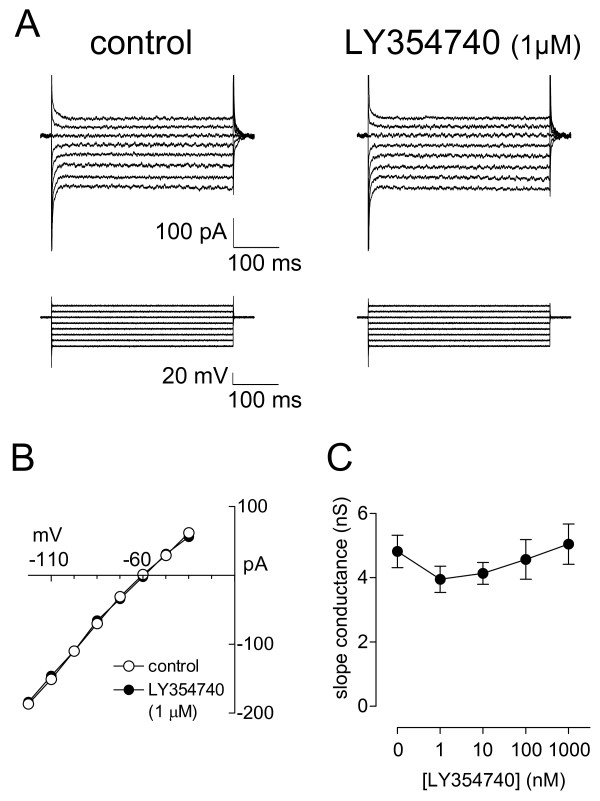
**Activation of group II mGluRs has no significant effect on current-voltage relationship and membrane slope conductance**. **A**: Voltage-clamp recordings of whole-cell currents elicited in one CeLC neuron by a series of 400 ms voltage steps (-110 to -40 mV) from a holding potential of -60 mV in control ACSF and during application of LY354740 (1 μM, 10 min). **B**: Current-voltage relationship of the same neuron as in A was calculated from the steady state current traces in A. **C**: Membrane slope conductance (in nS) is calculated from the current-voltage (I-V) relationships constructed from steady-state current traces like those shown in A in the presence and absence of the different concentrations of LY354740. In the sample of CeLC neurons (n = 13) LY354740 (1–1000 nM) had no significant effect on membrane slope conductance compared to predrug control "0" (P > 0.05, ANOVA with post-hoc Dunnett's test). Symbols and error bars represent mean ± SE. Neurons were held at -60 mV.

**Figure 5:**
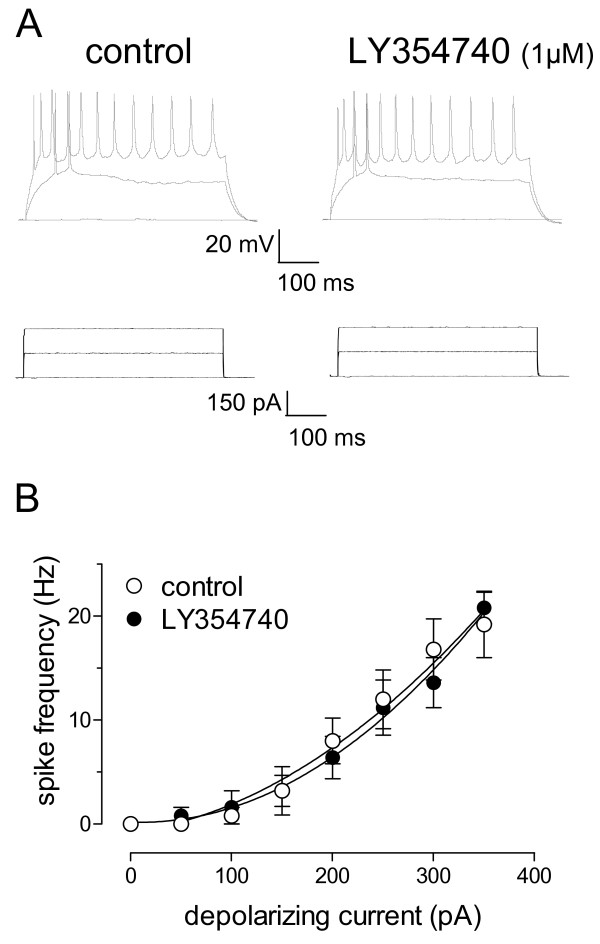
**LY354740 has no effect on neuronal excitability of CeLC neurons in the arthritis pain model**. **A**: Current-clamp recordings of action potentials (top) generated in a CeLC neuron by direct (through the patch electrode) intracellular injections of current pulses (500 ms; bottom) of increasing magnitude before and during LY354740 administration. LY354740 (1 μM, 10 min) did not change action potential firing rate. **B**: Action potential firing rate measured by intracellular injection of current pulses of increasing magnitude (50 pA steps) was not significantly changed in the presence of LY354740 (1 μM) (n = 6). Symbols and error bars represent mean ± SE. For the measurement of action potential firing in current clamp, neurons were recorded at -60 mV.

To determine the site of group II mGluR action in the CeLC we used a number of well-established electrophysiological methods, including the analysis of amplitude and frequency of spontaneous miniature EPSCs (mEPSCs) (Fig. 3), current-voltage relationships (Fig. 4) and neuronal excitability (Fig. 5). These parameters were measured before and during application of LY354740 in amygdala brain slices from arthritic rats.

The analysis of spontaneous mEPSCs in the presence of TTX is a well established electrophysiological approach to determine pre- versus post-synaptic mechanisms. Presynaptic changes at the transmitter release site affect mEPSC frequency, whereas changes at the postsynaptic membrane would alter mEPSC amplitude (quantal size) [8,33]. LY354740 decreased the frequency, but not amplitude, of mEPSCs recorded in TTX (1 μM)-containing ACSF in slices from arthritic rats (Fig. 3). This presynaptic effect is illustrated in the current traces recorded in voltage-clamp mode in an individual CeLC neuron (Fig. 3A). In the sample of neurons, LY354740 decreased the mean mEPSC frequency significantly (53.4 ± 12.8% of predrug, P < 0.05, paired t-test, n = 4; Fig. 3B). LY354740 had no effect on the amplitude of mEPSCs (103 ± 5.83% of predrug, P > 0.05, paired t-test, n = 4; Fig. 3C). In the absence of evidence for postsynaptic mechanisms at the concentration of maximal effect (1 μM), it is unlikely that any postsynaptic effects are caused by lower concentrations, at which the lower potency o fLY354740 under normal conditions compared to arthritis is revealed.

Concentrations of LY354740 that clearly decreased synaptic transmission did not change the membrane slope conductance measured from the current-voltage (I-V) relationships. Fig. 4A shows steady-state current traces elicited by a series of voltage steps in an individual CeLC neuron before (control) and during application of LY354740 (1 μM). The I-V relationship calculated from the steady state current traces shown in Fig. 4A did not change in the presence of LY354740 (1 μM; Fig. 4B). The data for the sample of neurons (n = 13) are summarized in Fig. 4C. LY354740 (1 nM – 1 μM) had no significant effect on the membrane slope conductances measured from the I-V relationships of individual neurons.

LY354740 had no significant effect on neuronal excitability (Fig. 5). Action potentials were evoked in current-clamp mode by direct intracellular depolarizing current injections (500 ms) of increasing magnitude through the patch electrode (Fig. 5A). Input-output functions of neuronal excitability were obtained by averaging the frequency of action potentials evoked at each current intensity (Fig. 5B). LY354740 (1 μM) had no significant effect on action potential ("spike") firing rate in neurons from arthritic rats.

Taken together, the significant reduction of mEPSC frequency, but not amplitude, and the lack of intrinsic membrane property changes in the presence of LY354740 suggest that group II mGluR-mediated inhibition of synaptic transmission occurs through a pre- rather than postsynaptic mechanism.

## Discussion

The present study is the first to show that group II mGluRs act as modulators to inhibit pain-related synaptic plasticity in the amygdala. The major findings of this study are as follows: (1) A selective group II mGluR agonist (LY354740) inhibits normal synaptic transmission in slices from normal rats and synaptic plasticity in neurons from arthritic rats. (2) LY354740 is more potent (25-fold) in CeLC neurons from animals with arthritis compared to control neurons from normal animals. (3) Antagonist (EGLU) studies show that the effects of LY354740 are receptor-mediated, but there is no evidence for intrinsic/endogenous receptor activation in slices. (4) The inhibitory effects of LY354740 on mEPSC frequency, but not amplitude, and the lack of effect on membrane properties suggest a pre- rather than postsynaptic mechanism of group II mGluR action in the CeLC.

The increased potency of LY354740 in neurons from arthritic rats could be explained by increased amount of receptor expression or increased receptor affinity for the agonist. Since the efficacy (maximum effect) of the agonist was not changed in the arthritis pain model, it is less likely that enhanced coupling efficiency to G-protein mediated second-messenger systems is involved. Group II mGluRs inhibit adenylyl cyclase via G_i_/G_o _proteins, resulting in decreased cyclic AMP (cAMP) formation and cAMP-dependent protein kinase (PKA) activation [25,26,30].

The underlying mechanism of increased group II mGluR function remains to be determined. Differential changes of synaptic inhibition by group II mGluRs in the amygdala (including the CeLC) have been shown in other models of neuroplasticity. Chronic cocaine treatment abolished the effects of group II mGluR agonists on synaptic transmission while increased potencies of group II mGluR agonists were observed in the kindling model of epilepsy [34,35]. Increased inhibition of synaptic transmission in the amygdala by group III mGluRs, which couple to similar signal transduction pathways as group II mGluRs, were observed in the arthritis pain model [23], following chronic cocaine [35] and in the kindling model of epilepsy [34,35]. These data suggest that group II mGluR function depends on the type or origin of neuroplasticity and is modulated differently than that of group III mGluRs.

The inhibitory effect of LY354740 was reversed by a selective and competitive group II mGluR antagonist (EGLU), confirming that the effects of LY354740 were receptor-mediated. Although higher agonist potency was observed in the arthritic pain model, the fact that EGLU itself had no significant effect on synaptic transmission would argue against any intrinsic/endogenous activation of these receptors *in vitro*. The absence of effects of EGLU on its own does not seem to be due to insufficient drug concentrations at the receptor because EGLU was able to reverse the inhibitory effect of the exogenously applied agonist (LY354740). Group II mGluRs show predominantly extra-synaptic localizations [24,25,36] such that they are not readily accessible by endogenous glutamate release from synaptic vesicles. Thus, antagonists would fail to produce any effects by themselves. Indeed, antagonist studies have reported a lack of endogenous activation of group II mGluRs for several brain areas [34,37,38]. Since the exogenous activation of group II mGluRs by LY354740 inhibited presynaptic neurotransmitter release more potently in the arthritic pain model than under normal conditions, group II mGluR agonists could be useful therapeutics for the management of arthritic pain and its emotional-affective component. Importantly, group II mGluRs are becoming intriguing drug targets for a variety of neuropsychiatric disorders, most notably anxiety and depression, which are intimately linked to pain [24,31].

The present study suggests a presynaptic mechanism of group II mGluR effects at the PB-CeLC synapse. Evidence for a presynaptic site also comes from in situ hybridization data showing a relatively high expression of group II mRNA in the parabrachial nucleus (origin of the PB-CeLC afferents) but not in the central nucleus [39,40]. Presynaptic inhibition by group II mGluRs has been observed in several different brain regions including synapses in the basolateral and central nuclei of the amygdala [34,35], substantia nigra pars compacta [41], hippocampal areas [42,43] and corticostriatal synapses [44].

Group II mGluRs have been implicated in nociceptive processing at different levels of the pain neuraxis, but their roles are less well understood than those of group I mGluRs. Activation of group II mGluRs in peripheral tissues had antinociceptive effects in models of inflammatory [27,45,46] and neuropathic pain [47]. Conversely, blockade of peripheral group II mGluRs (LY341495) prolonged PGE2- and carrageenan-induced mechanical allodynia, suggesting that peripheral group II mGluRs mediate endogenous anti-allodynic effects [46]. Similarly, peripheral injection of a group II/III antagonist [MSOPPE, (RS)-alpha-methylserine-O-phosphate monophenyl ester] enhanced glutamate-induced mechanical allodynia [27]. Activation of spinal group II mGluRs inhibited behavior [48,49] and central sensitization [50,51] related to inflammatory pain. Intracisternal and systemic, but not intrathecal, administration of group II agonists inhibited formalin-induced pain behavior [52,53]. Behavioral data suggest that group II mGluRs in the periaqueductal gray (PAG) inhibit descending facilitation of pain behavior thus activating descending pain inhibition [54]. Activation of group II in the ventrobasal thalamus resulted in the disinhibition of nociceptive processing through the presynaptic reduction of GABAergic inhibition [55].

## Methods

Male Sprague Dawley rats (90 g–190 g; mean 130 g) were housed in a temperature controlled room and maintained on a 12 h day/night cycle. Water and food were available *ad libitum*. Electrophysiological data were obtained from untreated normal rats and rats with monoarthritis in the knee (6 h after induction). All experimental procedures were approved by the Institutional Animal Care and Use Committee (IACUC) at the University of Texas Medical Branch (UTMB) and conform to the guidelines of the International Association for the Study of Pain (IASP) and of the National Institutes of Health (NIH).

### Arthritis pain model

In the group of arthritic rats, arthritis was induced in the left knee joint as previously described [8,19-21]. A kaolin suspension (4%, 100 μl) was injected into the left knee joint cavity through the patellar ligament. After repetitive flexions and extensions of the knee for 15 minutes, a carrageenan solution (2%, 100 μl) was injected into the knee joint cavity, and the leg was flexed and extended for another 5 minutes. Brain slices for the electrophysiological studies were obtained 6 hours after arthritis induction.

### Amygdala slice preparation

After decapitation, the brains were quickly dissected out and blocked in cold (4°C) artificial cerebrospinal fluid (ACSF) containing (in mM) 117 NaCl, 4.7 KCl, 1.2 NaH_2_PO_4_, 2.5 CaCl_2_, 1.2 MgCl_2_, 25 NaHCO_3_, and 11 glucose. ACSF was oxygenated and equilibrated to pH7.4 with a mixture of 95% O_2 _and 5% CO_2_. Coronal brain slices (500 μm) containing the CeLC were prepared using a Vibroslice (Camden instruments, London, UK). The slices were then incubated in ACSF at room temperature (21°C) for at least 1 h and a single brain slice was transferred to the recording chamber and submerged in ACSF (31 ± 1°C), which superfused the slice at ~2 ml/min.

### Whole-cell patch-clamp recording

Whole-cell patch-clamp recordings using the "blind" patch technique were obtained from CeLC neurons as described before [8,13,21,22]. Patch electrodes were made from 1.5 mm borosilicate glass capillaries (1.5 mm outer diameter, 1.12 mm inner diameter; Drummond, Broomall, PA) pulled on a Flaming-Brown micropipette puller (P-80/PC; Sutter Instrument Co., Novato, CA). Recording electrodes were positioned in the CeLC under visual control. The boundaries of the CeLC are easily discerned under light microscopy (see Fig. 8A in [8]); each slice was matched with the corresponding level in Paxinos and Watson (1998). The internal solution of the recording electrodes (4- to 6-MΩ tip resistance) contained (in mM) 122 K-gluconate, 5 NaCl, 0.3 CaCl_2_, 2 MgCl_2_, 1 EGTA, 10 HEPES, 5 Na_2_-ATP, and 0.4 Na_3_-GTP; pH was adjusted to 7.2–7.3 with KOH and the osmolarity to 280 mmol/kg with sucrose.

After tight (>1GΩ) seals were formed and the whole-cell configuration was obtained, neurons were included in the sample if the resting membrane potential was more negative than -50 mV and action potentials overshooting 0 mV were evoked by direct cathodal stimulation. Voltage and current signals were low-pass filtered at 1 kHz with a dual 4-pole Bessel filter (Warner Instrument Corp., Hamden, CT), digitized at 5 KHz (Digidata 1322 interface, Axon Instruments, Foster City, CA), and stored on a Pentium 4 computer (Dell). Data were also continuously recorded on a pen chart recorder (Gould 3400, Gould Instr., Valley View, OH). Evoked potential and evoked current data were acquired and analyzed using pCLAMP8 software (Axon Instruments, Foster City, CA). Discontinuous single-electrode voltage clamp (d-SEVC) recordings were acquired using an Axoclamp-2B amplifier (Axon Instruments) with a switching frequency of 5–6 kHz (30% duty cycle), gain of 3–8 nA/mV, and time constant of 20 ms. Phase shift and anti-alias filter were optimized. The headstage voltage was monitored continuously on a digital oscilloscope (Gould 400, Gould Instr., Modesto, CA) to ensure precise performance of the amplifier. Neurons were held at -60 mV.

### Synaptic stimulation

We studied transmission at the parabrachial area (PB)-CeLC synapse that provides nociceptive input to the CeLC from the spinal cord and brainstem (see Introduction). Using a concentric bipolar stimulating electrode (Kopf Instruments) of 22 kΩ resistance, excitatory postsynaptic currents (EPSCs) were evoked in CeA neurons by electrical stimulation (using a Grass S88 stimulator, Grass Instr.) of the PB-CeLC synapse. The stimulation electrode was positioned under microscopic control on the fibers dorsomedial to the CeA and ventral to but outside of the caudate-putamen [8,21,22] (see Fig. 8A in [8]). Electrical stimuli (150 μs square-wave pulses) were delivered at frequencies below 0.25 Hz. Input-output relations were obtained by increasing the stimulus intensity in 100 μA steps. For evaluation of a drug effect on synaptically evoked responses, the stimulus intensity was adjusted to 80% of the intensity required for orthodromic spike generation, which was on average 350–450 μA.

### Drugs

The following drugs were used: (S)-alpha-ethylglutamic acid (EGLU; selective group II mGluR antagonist [25,32], purchased from Tocris Cookson Inc., Ellisville, MO; (+)-2-aminobicyclo [3.1.0] hexane-2,6-dicarboxylic acid (LY354740; selective group II mGluR agonist [24,31], a generous gift from Eli Lilly and Company. All drugs were dissolved in ACSF and applied by gravity-driven superfusion of the slice in ACSF. Solution flow into the recording chamber (1 ml volume) was controlled with a three-way stopcock. Drug applications were for at least 10 min in duration to establish equilibrium in the tissue.

### Data analysis and statistics

All averaged values are given as the mean ± SE. Statistical significance was accepted at the level of P < 0.05. Concentration-response relationships were compared using a two-way analysis of variance (ANOVA). IC_50 _values were calculated from sigmoid curves fitted to the cumulative concentration-response data by nonlinear regression using the formula *y *= *A *+ (*B *- *A*)/[*1 *+ (*10*^*C*^/*10*^*X*^)^*D*^], where A = bottom plateau, B = top plateau, C = log(IC_50_), D = slope coefficient (GraphPad Prism 3.0). Using the linear curve fit function of pClamp software (Axon Instruments), membrane slope conductances (in nS) in the absence and presence of agonists were calculated from the linear portion of the current-voltage (I-V) relationships recorded in voltage-clamp mode. An ANOVA with post-hoc Dunnett's test was used to compare the drug effects on membrane slope conductance to the predrug control value. Drug effects (agonist and antagonist) were compared to predrug control values using the paired t-test. mEPSCs were analyzed for frequency and amplitude distributions using the MiniAnalysis program 5.3 (Synaptosoft Inc., Decatur, GA). The detection threshold was set to 3 times the average RMS noise level during event-free sections (typically 10 pA; see Fig. 3C). The analysis was re-checked manually. The Kolmogorov-Smirnov test was used for the cumulative distribution analysis of mEPSC amplitude and frequency while the paired t-test compared mean amplitude and frequency of mEPSCs.

## Conclusion

Activation of presynaptic group II mGluRs in the CeLC inhibits synaptic plasticity in a model of arthritis pain more potently than normal synaptic transmission, suggesting that presynaptic group II mGluRs in the CeLC may be potential therapeutic targets for pain relief.

## Authors' contributions

JSH, YF and GCB carried out the experiments and performed the data analysis. VN conceptualized the hypothesis, designed, participated in and supervised the experiments and directed the data analysis. JSH drafted the manuscript and VN revised the manuscript. All authors read and approved the final manuscript.

## References

[B1] Phelps EA, Ledoux JE (2005). Contributions of the Amygdala to Emotion Processing: From Animal Models to Human Behavior. Neuron.

[B2] Davidson RJ (2002). Anxiety and affective style: role of prefrontal cortex and amygdala. Biol Psychiatry.

[B3] Maren S (2005). Synaptic Mechanisms of Associative Memory in the Amygdala. Neuron.

[B4] Gallagher RM, Verma S (2004). Mood and anxiety disorders in chronic pain. Prog in Pain Res Management.

[B5] Neugebauer V, Li W, Bird GC, Han JS (2004). The amygdala and persistent pain. The Neuroscientist.

[B6] Hebert MA, Ardid D, Henrie JA, Tamashiro K, Blanchard DC, Blanchard RJ (1999). Amygdala lesions produce analgesia in a novel, ethologically relevant acute pain test. Physiol Behav.

[B7] Nandigama P, Borszcz GS (2003). Affective analgesia following the administration of morphine into the amygdala of rats. Brain Res.

[B8] Han JS, Li W, Neugebauer V (2005). Critical role of calcitonin gene-related peptide 1 receptors in the amygdala in synaptic plasticity and pain behavior. J Neurosci.

[B9] Bornhovd K, Quante M, Glauche V, Bromm B, Weiller C, Buchel C (2002). Painful stimuli evoke different stimulus-response functions in the amygdala, prefrontal, insula and somatosensory cortex: a single-trial fMRI study. Brain.

[B10] Bonaz B, Baciu M, Papillon E, Bost R, Gueddah N, Le Bas JF, Fournet J, Segebarth C (2002). Central processing of rectal pain in patients with irritable bowel syndrome: an fMRI study. Am J Gastroenterol.

[B11] Schneider F, Habel U, Holthusen H, Kessler C, Posse S, Muller-Gartner HW, Arndt JO (2001). Subjective ratings of pain correlate with subcortical-limbic blood flow: an fMRI study. Neuropsychobiology.

[B12] Bingel U, Quante M, Knab R, Bromm B, Weiller C, Buchel C (2002). Subcortical structures involved in pain processing: evidence from single-trial fMRI. Pain.

[B13] Gauriau C, Bernard J-F (2002). Pain pathways and parabrachial circuits in the rat. Exp Physiol.

[B14] Jasmin L, Burkey AR, Card JP, Basbaum AI (1997). Transneuronal labeling of a nociceptive pathway, the spino-(trigemino-)parabrachio-amygdaloid, in the rat. J Neurosci.

[B15] Braz JM, Nassar MA, Wood JN, Basbaum AI (2005). Parallel "pain" pathways arise from subpopulations of primary afferent nociceptor. Neuron.

[B16] LeDoux JE (2000). Emotion circuits in the brain. Ann Rev Neuroscience.

[B17] Heinricher MM, McGaraughty S, Lydic R, Baghdoyan HA (1999). Pain-modulating neurons and behavioral state. Handbook of Behavioral State Control.

[B18] Neugebauer V, Li W (2003). Differential sensitization of amygdala neurons to afferent inputs in a model of arthritic pain. J Neurophysiol.

[B19] Li W, Neugebauer V (2004). Differential roles of mGluR1 and mGluR5 in brief and prolonged nociceptive processing in central amygdala neurons. J Neurophysiol.

[B20] Li W, Neugebauer V (2004). Block of NMDA and non-NMDA receptor activation results in reduced background and evoked activity of central amygdala neurons in a model of arthritic pain. Pain.

[B21] Neugebauer V, Li W, Bird GC, Bhave G, Gereau RW (2003). Synaptic plasticity in the amygdala in a model of arthritic pain: differential roles of metabotropic glutamate receptors 1 and 5. J Neurosci.

[B22] Bird GC, Lash LL, Han JS, Zou X, Willis WD, Neugebauer V (2005). PKA-dependent enhanced NMDA receptor function in pain-related synaptic plasticity in amygdala neurons. J Physiol.

[B23] Han JS, Bird GC, Neugebauer V (2004). Enhanced group III mGluR-mediated inhibition of pain-related synaptic plasticity in the amygdala. Neuropharmacology.

[B24] Swanson CJ, Bures M, Johnson MP, Linden AM, Monn JA, Schoepp DD (2005). Metabotropic glutamate receptors as novel targets for anxiety and stress disorders. Nat Rev Drug Discov.

[B25] Schoepp DD, Jane DE, Monn JA (1999). Pharmacological agents acting at subtypes of metabotropic glutamate receptors. Neuropharmacology.

[B26] Neugebauer V (2001). Metabotropic glutamate receptors: novel targets for pain relief. Exp Rev Neurother.

[B27] Neugebauer V, Carlton SM (2002). Peripheral metabotropic glutamate receptors as drug targets for pain relief. Expert Opin on Ther Targets.

[B28] Lesage ASJ (2004). Role of group I metabotropic glutamate receptors mGlu1 and mGlu5 in nociceptive signalling. Curr Neuropharm.

[B29] Fundytus ME (2001). Glutamate receptors and nociception. Implications for the drug treatment of pain. CNS Drugs.

[B30] Varney MA, Gereau RW (2002). Metabotropic glutamate receptor involvement in models of acute and persistent pain: prospects for the development of novel analgesics. Current Drug Targets.

[B31] Marek GJ (2004). Metabotropic glutamate 2/3 receptors as drug targets. Curr Opin Pharmacol.

[B32] Jane DE, Thomas NK, Tse HW, Watkins JC (1996). Potent antagonists at the L-AP4- and (1S,3S)-ACPD-sensitive presynaptic metabotropic glutamate receptors in the neonatal rat spinal cord. Neuropharmacology.

[B33] McKernan MG, Shinnick-Gallagher P (1997). Fear conditioning induces a lasting potentiation of synaptic currents in vitro. Nature.

[B34] Neugebauer V, Keele NB, Shinnick-Gallagher P (1997). Epileptogenesis in vivo enhances the sensitivity of inhibitory presynaptic metabotropic glutamate receptors in basolateral amygdala neurons in vitro. J Neurosci.

[B35] Neugebauer V, Zinebi F, Russell R, Gallagher JP, Shinnick-Gallagher P (2000). Cocaine and kindling alter the sensitivity of group II and III metabotropic glutamate receptors in the central amygdala. J Neurophysiol.

[B36] Cartmell J, Schoepp DD (2000). Regulation of neurotransmitter release by metabotropic glutamate receptors. J Neurochem.

[B37] Bushell TJ, Jane DE, Tse HW, Watkins JC, Garthwaite J, Collingridge GL (1996). Pharmacological antagonism of the actions of group II and III mGluR agonists in the lateral perforant path of rat hippocampal slices. Br J Pharmacol.

[B38] Salt TE, Eaton SA, Turner JP (1996). Characterization of the metabotropic glutamate receptors (mGluRs) which modulate GABA-mediated inhibition in the ventrobasal thalamus. Neurochem Int.

[B39] Ohishi H, Shigemoto R, Nakanishi S, Mizuno N (1993). Distribution of the mRNA for a metabotropic glutamate receptor (mGluR3) in the rat brain: an in situ hybridization study. J Comp Neurol.

[B40] Ohishi H, Shigemoto R, Nakanishi S, Mizuno N (1993). Distribution of the messenger RNA for a metabotropic glutamate receptor, mGluR2, in the central nervous system of the rat. Neuroscience.

[B41] Wang L, Kitai ST, Xiang Z (2005). Modulation of excitatory synaptic transmission by endogenous glutamate acting on presynaptic group II mGluRs in rat substantia nigra compacta. J Neurosci Res.

[B42] Price CJ, Karayannis T, Pal BZ, Capogna M (2005). Group II and III mGluRs-mediated presynaptic inhibition of EPSCs recorded from hippocampal interneurons of CA1 stratum lacunosum moleculare. Neuropharmacology.

[B43] Manzoni OJ, Castillo PE, Nicoll RA (1995). Pharmacology of metabotropic glutamate receptors at the mossy fiber synapses of the guinea pig hippocampus. Neuropharmacology.

[B44] Lovinger DM, McCool BA (1995). Metabotropic glutamate receptor-mediated presynaptic depression at corticostriatal synapses involves mGLuR2 or 3. J Neurophysiol.

[B45] Yang D, Gereau RW (2002). Peripheral Group II Metabotropic Glutamate Receptors (mGluR2/3) Regulate Prostaglandin E2-Mediated Sensitization of Capsaicin Responses and Thermal Nociception. J Neurosci.

[B46] Yang D, Gereau RW (2003). Peripheral group II metabotropic glutamate receptors mediate endogenous anti-allodynia in inflammation. Pain.

[B47] Jang JH, Kim DW, Sang Nam T, Se Paik K, Leem JW (2004). Peripheral glutamate receptors contribute to mechanical hyperalgesia in a neuropathic pain model of the rat. Neuroscience.

[B48] Dolan S, Nolan AM (2002). Behavioral evidence supporting a differential role for spinal group I and II metabotropic glutamate receptors in inflammatory hyperalgesia in sheep. Neuropharmacology.

[B49] Soliman AC, Yu JSC, Coderre TJ (2005). mGlu and NMDA receptor contributions to capsaicin-induced thermal and mechanical hypersensitivity. Neuropharmacology.

[B50] Neugebauer V, Chen P-S, Willis WD (2000). Groups II and III metabotropic glutamate receptors differentially modulate brief and prolonged nociception in primate STT cells. J Neurophysiol.

[B51] Stanfa LC, Dickenson AH (1998). Inflammation alters the effects of mGlu receptor agonists on spinal nociceptive neurones. Eur J Pharmacol.

[B52] Jones CK, Eberle EL, Peters SC, Monn JA, Shannon HE (2005). Analgesic effects of the selective group II (mGlu2/3) metabotropic glutamate receptor agonists LY379268 and LY389795 in persistent and inflammatory pain models after acute and repeated dosing. Neuropharmacology.

[B53] Simmons RM, Webster AA, Kalra AB, Iyengar S (2002). Group II mGluR receptor agonists are effective in persistent and neuropathic pain models in rats. Pharmacol Biochem and Behav.

[B54] Maione S, Oliva P, Marabese I, Palazzo E, Rossi F, Berrino L, Rossi F, Filippelli A (2000). Periaqueductal gray matter metabotropic glutamate receptors modulate formalin-induced nociception. Pain.

[B55] Salt TE (2002). Glutamate receptor functions in sensory relay in the thalamus. Philos Trans R Soc of Lond B, Biol Sci.

